# Constructing three-way classifier with interval granulation neighborhood rough sets based on uncertainty invariance

**DOI:** 10.3389/fnbot.2026.1787501

**Published:** 2026-04-01

**Authors:** Yongqi Wang, Taihua Xu, Rong Huang, Zhuangzhuang Liu, Jie Yang

**Affiliations:** 1 School of Computer, Jiangsu University of Science and Technology, Zhenjiang, Jiangsu, China; 2 School of Physics and Electronic Science, Zunyi Normal University, Zunyi, Guizhou, China

**Keywords:** classifier, DBSCAN, fuzziness loss, interval granulation neighborhood rough sets, three-way decision

## Abstract

Three-way decision with neighborhood rough sets (3WDNRS) is effective in handling uncertain problems involving continuous data through the adjustment of the neighborhood radius. However, it faces two main limitations. Firstly, 3WDNRS relies on individual neighborhood granules as inputs, which can impair both decision efficiency and model generalizability. Secondly, the thresholds used in 3WDNRS often require predefinition based on prior knowledge, making the method difficult to apply in situations where such knowledge is lacking. To address these problems, this study introduces interval granulation (IG) into 3WD to construct an effective three-way classifier. Firstly, an interval granulation method based on DBSCAN is proposed. Then, an interval granulation neighborhood rough sets (IGNRS) model is presented, combining IG with quality indicators. Based on the IGNRS, a three-way classifier called 3WD-IGNRS is proposed by considering the principle of minimum fuzzy loss. Finally, extensive comparative experiments are conducted with three state-of-the-art granular-ball (GB)-based classifiers and four classical machine learning classifiers on 12 public benchmark datasets. The results demonstrate that our models consistently outperform the compared methods, achieving an average accuracy improvement of 4.94% compared to the best-performing granular-ball classifier.

## 1 Introduction

Decision-making under uncertainty is a fundamental challenge in artificial intelligence and data mining. Three-way decision (3WD) (Yao, 2018, 2020, 2023), originally proposed by Yao, provides an effective paradigm for addressing such complexity. Unlike traditional binary models that force an immediate acceptance or rejection, 3WD introduces a deferred decision mechanism, partitioning the universe into positive, negative, and boundary regions. This flexibility effectively reduces decision risks and has been widely applied in various domains. Recent studies have further expanded the boundaries of uncertainty handling. For instance, Singh and Huang (2023) developed novel models based on ambiguous set theory for complex decision-making problems, such as college selection and sunspot forecasting (Singh, 2025b). Furthermore, theoretical extensions regarding single-valued ambiguous numbers and aggregation operators have significantly enriched the mathematical foundations for handling vague information (Singh, 2025a, 2023).

Despite these theoretical successes, applying 3WD to large-scale continuous data remains challenging. While Neighborhood Rough Sets (NRS) (Hu et al., 2008) effectively handle continuous attributes, they typically rely on fine-grained, sample-level granules, which leads to high computational complexity and poor noise tolerance. To address efficiency issues, Granular-Ball Computing (GBC) (Xia et al., 2019) was proposed to represent data using coarse-grained hyperspherical granules. However, the strict geometric assumption of hyperspherical balls in standard GBC often fails to cover irregular data boundaries accurately, leading to information loss and “fuzziness gaps" at decision boundaries.

Motivated by these limitations, this study proposes a novel framework that integrates Interval Granulation (IG) with Three-Way Decision. Unlike traditional GBC, our approach utilizes a density-based interval granulation method (DBIG) using DBSCAN (Cheng et al., 2024) to flexibly cover the data space, effectively preserving boundary information. Furthermore, we introduce an uncertainty invariance principle to objectively determine the decision thresholds, minimizing the fuzziness loss during the granular transformation process.

The main contributions of this study are summarized as follows:

(1) An interval granulation method based on DBSCAN, termed DBIG, is proposed to reduce uncertainty caused by inaccurate cluster center selection and rigid geometric assumptions.
(2) A quality evaluation metric for interval granules is developed, based on which an improved interval granulation neighborhood rough sets (IGNRS) model is constructed.
(3) By integrating three-way decision theory with IGNRS, a three-way classifier minimizing fuzzy loss, named 3WD-IGNRS, is proposed. This model avoids subjective risk parameter specification and exhibits stronger generalization capability.

The remainder of this study is organized as follows. Section 2 reviews related work. Section 3 presents the proposed IGNRS model. Section 4 introduces the construction of the three-way decision framework. Section 5 reports experimental results. Finally, Section 6 concludes the study.

## 2 Related work

### 2.1 Three-way decision and neighborhood rough sets

Three-way decision (3WD) theory has evolved significantly to handle uncertainty in information systems, often being investigated in conjunction with rough sets (Zou et al., 2023), fuzzy sets (Yang and Yao, 2021), and concept lattices (Xie et al., 2025).

Particularly, the integration of 3WD with granular computing has emerged as a highly promising research direction for robust decision-making. Recent breakthroughs by Yang et al. have deeply integrated 3WD with granular-ball structures using the framework of shadowed sets. For instance, they proposed a cost-sensitive three-way granular-ball generation (CS3W-GBG) method to optimize decision risks (Yang et al., 2025). To handle dynamic data environments, they further developed a three-way incremental granular-ball classifier based on shadowed sets (Yang et al., 2026b). Moreover, this shadowed granular-ball paradigm has been successfully extended to effectively identify complex anomalies in outlier detection tasks (Yang et al., 2026a). Alongside these developments, other granular perspectives have also been explored; for example, Tang et al. (2025) proposed a three-way class-specific attribute reducts framework, and Yu et al. (2024) developed a three-way graph convolutional neural network.

Neighborhood Rough Sets (NRS) (Hu et al., 2008; Zhang et al., 2024; Nguyen et al., 2025) extend classical rough set theory by replacing equivalence relations with covering relations, providing a neighborhood-based granular foundation. Recent advancements in fuzzy-rough set models have focused on optimizing feature selection and dimensionality reduction. Tiwari et al. (2024) introduced a hybrid similarity relation-based mutual information for feature selection in intuitionistic fuzzy rough frameworks. Similarly, Shreevastava et al. (2025) proposed an (α, β)-indiscernibility-assisted model for dimensionality reduction, while Jain et al. (2023) addressed missing value imputation combined with feature selection. Additionally, tolerance-based approaches for attribute reduction have been explored to enhance model robustness (Tiwari et al., 2018).

Despite these theoretical advancements and the successful integration of 3WD with granular balls, existing 3WD models often rely on strict geometric hyperspheres or fine-grained individual samples as basic decision units, which can restrict their flexibility in covering irregular boundaries. Furthermore, threshold determination still requires sophisticated optimization. This motivates our exploration into interval granulation.

### 2.2 Granular-ball computing and its variants

Granular-ball computing (GBC) (Xia et al., 2019, 2021, 2022) is an efficient granulation paradigm that adaptively generates granular balls (GBs) using clustering strategies. GBC has been successfully applied in anomaly detection (Li et al., 2025; Gao et al., 2025), clustering (Xia et al., 2025; Su et al., 2025), and classification (Liu et al., 2025). By replacing traditional information granules with GBs, GBC significantly improves computational efficiency. Xia et al. (2020) further integrated GBC with NRS to propose granular-ball neighborhood rough sets (GBNRS).

However, despite these improvements in efficiency, most existing GBC models primarily rely on **hyperspherical structures**, which impose strict geometric symmetry. This assumption restricts their ability to flexibly cover irregular data distributions, leading to inadequate handling of fuzziness at decision boundaries. Furthermore, random initialization or the use of sample means for cluster centers introduces instability. To overcome these issues, Wu et al. (2024) proposed an interval granulation method based on the uncertainty principle. Building on this, our study introduces a density-based approach to ensure interval granule centers align better with intrinsic data distributions.

## 3 Preliminaries

In this section, we review some essential definitions to facilitate understanding of the framework presented in this study. The key symbols used in this study are summarized in Table 1.

**Table 1:** Key symbols and their descriptions.

Symbol	Description
*U*	The non-empty finite universe of samples
*A*	The set of conditional attributes
*d*	The decision attribute
*V*_*a*_	The domain of attribute *a*∈*A*
*IG*	An interval granule
*IGs*	The set of all interval granules generated by DBIG
${ℐ}_{R}$	Interval-induced equivalence relation
ρ(*x*)	Local density of sample *x* based on DBSCAN
*C*_ρ_	Granularity center based on density
*m*(*IG*)	Quality indicator (membership degree) of an interval granule
α, β	The pair of thresholds for three-way decision (α>β)
α^*^, β^*^	The optimal threshold pair obtained by minimizing fuzziness
*H*(*X*)	The fuzziness (entropy) of a fuzzy set *X*
*POS, BND, NEG*	Positive, Boundary, and Negative regions

^*^ denotes the optimal value.

Let *NS* = (*U, A*∪*d, V, f*) be a neighborhood decision system, where *U* = {*x*_1_, *x*_2_, ⋯ , *x*_*n*_} is a non-empty finite set. In detail, *A* is the conditional attribute set, *a*∈*A* is a conditional attribute, and *d* is the decision attribute set. *V* = *V*_*A*_∪*V*_*d*_, *V*_*A*_ = ⋃_*a*∈*A*_*V*_*a*_, *V*_*a*_ = {*f*(*x, a*)|*x*∈*U*} and *V*_*d*_ = {*f*(*x, d*)|*x*∈*U*} are the domain of *a* and *d*, respectively. *f*:*U*×(*A*∪{*d*}) → *V* is a mapping function.

**Definition 1** [Interval granulation of attribute values (Wu et al., 2024)] Given a neighborhood decision system *NS* = (*U, A*∪*d, V, f*), *a*∈*A* is a numerical attribute. *V*_*a*_ is the domain of *a*. An interval granulation on attribute *V*_*a*_ is defined as follows:


$$\begin{array}{l} I_{a}=\left\{I_{a}^{1},I_{a}^{2},\ldots ,I_{a}^{q_{a}}\right\} \end{array}$$ (1)

where $I_{a}^{i}=\left[{\underline{V}}_{a}^{i},\bar{V_{a}^{i}}\right]$ ( *i* = 1, 2, …, *q*_*a*_ ) is the *i*-th interval of *a*, *q*_*a*_ is the number of interval of *a*, and *q*_*a*_ ≤ |*V*_*a*_|. ${\underline{V}}_{a}^{i}$ and $\bar{V_{a}^{i}}$ are the left and right boundaries of interval $I_{a}^{i}$, respectively. They can also be collectively described as the breakpoints of the numerical attribute *a*. Interval granulation of *V*_*a*_ satisfies three conditions as follows:

1. ${\underline{V}}_{a}^{i}\in V_{a},\bar{V_{a}^{i}}\in V$;
2. $V_{a}^{\min}=\underline{V_{a}^{1}}\leq \bar{V_{a}^{1}}<\underline{V_{a}^{2}}\leq \bar{V_{a}^{2}}<,\cdots ,<\underline{V_{a}^{q_{a}}}\leq \bar{V_{a}^{q_{a}}}=V_{a}^{\max},V_{a}^{\min}$ and $V_{a}^{\text{max}}$ are respectively the minimum and maximum values of *V*_*a*_;
3. For every *x*∈*U* and *a*∈*A*, there exists $I_{a}^{i}=\left[\underline{V_{a}^{i}},\bar{V_{a}^{i}}\right]$, such that ${\underline{V}}_{a}^{i}\leq f(x,a)\leq \bar{V_{a}^{i}}$.

**Figure 1:**
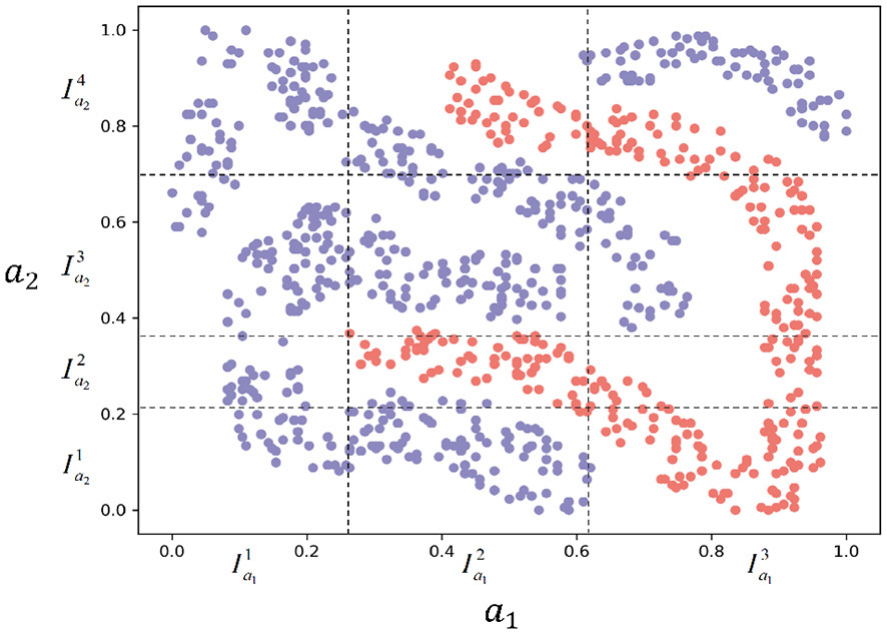
*Fourclasses* is divided into 12 subspaces induced by *I*_*a*_1__ and *I*_*a*_2__.

To facilitate a more intuitive understanding of Definition 1, we use the UCI dataset “fourclass" as an example. Treating this dataset as a neighborhood decision system, *a*_1_ and *a*_2_ represent two numerical attributes, respectively. As shown in Figure 1, the horizontal axis represents attribute *a*_1_, while the vertical axis denotes attribute *a*_2_. The domain of attribute *a*_1_ is divided into three intervals ($I_{a_{1}}^{1}$, $I_{a_{1}}^{2}$, $I_{a_{1}}^{3}$), while the domain of attribute *a*_2_ is divided into four intervals ($I_{a_{2}}^{1}$, $I_{a_{2}}^{2}$, $I_{a_{2}}^{3}$, $I_{a_{2}}^{4}$). Therefore, 12 subspaces are induced by *I*_*a*_1__ and *I*_*a*_2__, as shown by the 12 rectangular regions in Figure 1.

**Definition 2** [Interval-induced equivalence relation (Wu et al., 2024)] Given a neighborhood decision system *NS* = (*U, A*∪*d, V, f*), *R*⊆*A*. Then the interval equivalence relation ${ℐ}_{R}$ induced by *R* on *U* is defined as follows:


$$\begin{array}{l} {ℐ}_{R}=\left\{\left(x,y\right)\in U^{2}|\forall_{a\in R}\left(f\left(x,a\right)\in I_{a}^{i}\wedge f\left(y,a\right)\in I_{a}^{i}\right)\right\} \end{array}$$ (2)

Where $I_{a}^{i}\in I_{a},i=1,2,\ldots ,q_{a}$, as shown in Equation 1. The mathematical formulations used in this study are presented in Equations 2–4, 6–10, 13, 15, 17–22, and 24, 25.

Combining Definition 1 and 2 above, it follows that if $\left(x,y\right)\in {ℐ}_{R}$, then all values of *x* and *y* under ∀_*a*∈*R*_ belong to the same interval, thereby satisfying the interval equivalence relation. When interval under attribute *a* satisfy $\underline{V_{a}^{i}}=\bar{V_{a}^{i}}$, the interval equivalence relation ${ℐ}_{a}$ degrades to the indistinguishable relation. Therefore, the interval equivalence relation is a generalization of the indistinguishable relation.

**Definition 3** [Interval granulation, IG (Wu et al., 2024)] Given a non-empty finite set *U* = {*x*_1_, *x*_2_, ⋯ , *x*_*n*_}. Let ${ℐ}_{R}$ induce a partition of *U* denoted as: *IGs* = {*IG*_1_, *IG*_2_, …, *IG*_*m*_}, where *m* represent the total number of interval granulation. For ∀*IG*_*j*_∈*IGs*(*j* = 1, 2, …, *m*), it is defined as follows:


$$\begin{array}{l} IG_{j}=\left\{x\in U|\forall a\in R,f\left(x,a\right)\in I_{a}^{i_{a}}\right\} \end{array}$$ (3)

Where $I_{a}^{i}\subseteq I_{a}$, *i* = 1, 2, …, *q*_*a*_, as shown in Equation 1.

**Definition 4** [Label and purity of IG (Wu et al., 2024)] Given a non-empty finite set *U* = {*x*_1_, *x*_2_, ⋯ , *x*_*n*_}. Let ${ℐ}_{R}$ induce a partition of *U* denoted as: *IGs* = {*IG*_1_, *IG*_2_, …, *IG*_*m*_}, where *m* represents the total number of interval granulation. For ∀*IG*_*j*_∈*IGs*, the label *l*(*IG*_*j*_) and purity *p*(*IG*_*j*_) of interval granulation *IG*_*j*_ are respectively defined as follows:


$$\begin{array}{l} \begin{array}{c} l\left(IG_{j}\right)=\underset{l\in V_{d}}{\text{arg max}}|\left\{x\in IG_{j}|f\left(x,d\right)=l\right\}| \end{array} \end{array}$$ (4)

$$\begin{array}{l} p\left(IG_{j}\right)=\frac{\left|\left\{x\in IG_{j}|f(x,d)=l\left(IG_{j}\right)\right\}\right|}{\left|IG_{j}\right|} \end{array}$$ (5)

where *V*_*d*_ is the domain of decision attribute *d*. *l*(*IG*_*j*_) is the label with the highest frequency in *IG*_*j*_, *p*(*IG*_*j*_) is the ratio of samples with label *l*(*IG*_*j*_) in *IG*_*j*_.

**Definition 5** [Neighborhood Rough Set (Hu et al., 2008)] Given a neighborhood decision system *NS* = (*U, A*∪*d, V, f*), *R*⊆*A* and *X*⊆*U*. The lower and upper approximations of *X* with respect to *R* are defined as follows:


$$\begin{array}{l} \underline{NR}(X)=\left\{x|\delta_{R}(x)\subseteq X,x\in U\right\} \end{array}$$ (6)

$$\begin{array}{l} \bar{NR}(X)=\left\{x|\delta_{R}(x)\cap X\neq \emptyset ,x\in U\right\} \end{array}$$ (7)

where δ represents the neighborhood radius, δ_*R*_(*x*) = {*x*∣Δ(*x, x*_*k*_) ≤ δ, *x*_*k*_∈*U*} and Δ(*x, x*_*k*_) denotes the distance between *x* and *x*_*k*_.

**Definition 6** [Average fuzzy set (Zhang et al., 2013)] Let *R*⊆*A* and *X*⊆*U*. *U*/*R* = {*g*_1_, *g*_2_, ⋯ , *g*_*m*_} is an approximate space on *U*. μ(*x*) denotes the membership of a sample *x*, where *x*∈*g*_*i*_(*i* = 1, 2, ⋯ , *m*). Then the average membership degree of *x* is expressed as follows:


$$\begin{array}{l} \bar{\mu }\left(x\right)=\bar{\mu }\left(g_{i}\right)=\frac{\sum_{x\in g_{i}}\mu \left(x\right)}{\left|g_{i}\right|} \end{array}$$ (8)

Where |*g*_*i*_| represents the number of samples included in *g*_*i*_. Then, the average fuzzy set of *X* is represented as follows:


$$\begin{array}{l} X_{R}^{J}=\frac{\bar{\mu }\left(g_{1}\right)}{g_{1}}+\frac{\bar{\mu }\left(g_{2}\right)}{g_{2}}+\cdots +\frac{\bar{\mu }\left(g_{m}\right)}{g_{m}}. \end{array}$$ (9)

According to Definition 6, the average membership degree of interval granulation *IG*_*j*_ can be easily derived as follows:


$$\begin{array}{l} \bar{\mu }\left(IG_{j}\right)=\frac{\underset{x\in IG_{j}}{\sum }\mu \left(x\right)}{\left|IG_{j}\right|} \end{array}$$ (10)

**Definition 7** [Fuzziness (Deluca and Termini, 1971)] Let *M* and *N* denote two different fuzzy subsets on *U*, and the *F*(*U*) be the family of all fuzzy sets on *U*. Let a mapping *H*:*F*(*U*) → [0, 1] denote the fuzziness of fuzzy subsets. The function *H* must satisfy the following conditions:

1. *H*(*M*) = 0, iff *M*∈*P*(*U*),where *P*(*U*) is a power set of *U*;
2. *H*(*M*) = 1, iff ∀*x*_*i*_∈*U*, $M\left(x_{i}\right)=\frac{1}{2}$;
3. *H*(*N*) ≤ *H*(*M*), iff ∀*x*_*i*_∈*U*,$N\left(x_{i}\right)\geq M\left(x_{i}\right)\geq \frac{1}{2}$ or $N\left(x_{i}\right)\leq M\left(x_{i}\right)\leq \frac{1}{2}$.

**Definition 8** [Average fuzziness (Zhang et al., 2013)] Let *R*⊆*A*, *X*⊆*U*, and $h\left(x\right)=4\bar{\mu }\left(x\right)\left(1-\bar{\mu }\left(x\right)\right)$. Then the average fuzziness of average fuzzy set $X_{R}^{J}$ is defined as follows:


$$\begin{array}{l} H_{X_{R}^{J}}=\frac{1}{\left|U\right|}\underset{x\in U}{\sum }h\left(x\right) \end{array}$$ (11)

## 4 An improved interval granulation neighborhood rough sets

**Figure 2:**
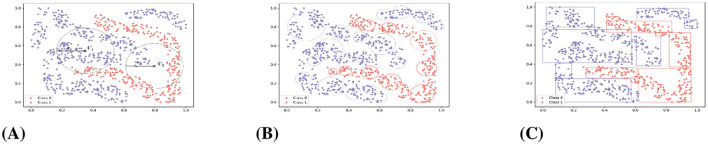
Output of GBC and IGC algorithm on the “fourclass" dataset. **(A)** The first iteration of GBC. **(B)** The final iteration of the GBC. **(C)** The final iteration of the IG. Horizontal and vertical coordinates denote sample attribute values. Different colors indicate different labels.

There are three limitations in the current GBC: loss of boundary information, random selection of granularity centers, and inability to adaptively adjust granularity size. As shown in Figure 2A, if the cluster center *C*_2_ that is located in the boundary region and distant from the high-density area is selected, the model's robustness is compromised due to its susceptibility to adjacent samples of the other class. Secondly, as shown in Figure 2B, after the final iteration of the GBC algorithm, it is evident that the GBs fail to fully cover the data space, which will result in the loss of some boundary information. As shown in Figure 2C, the interval granulation method improves boundary coverage. To address the above issues, Wu et al. (2024) proposed a data-driven interval granulation method that employs purity to evaluate the quality of interval granulation.

However, relying solely on the purity metric presents two problems: (1) During granularity center selection, it is challenging to determine the suitability of samples as granularity centers; (2) The purity metric cannot comprehensively evaluate the robustness, validity, and stability of interval granulation. In order to solve these problems, we first propose a density-based clustering algorithm based on DBSCAN to optimize interval granulation center selection, and then introduce a quality metric to evaluate its quality. Furthermore, based on the generated interval granulation and the quality metric, we define an improved interval granulation neighborhood rough sets (IGNRS).

### 4.1 Interval granulation generation based on DBSCAN

Unlike partitioning and hierarchical clustering algorithms, DBSCAN effectively divides high-density regions into clusters by identifying them as the maximum density connected point sets, and can recognize clusters of arbitrary shapes in spatial databases containing noise. Specifically, first process the dataset using the DBSCAN algorithm to assign samples to different clusters. Then calculate the local density of the samples. Finally, select the cluster centers based on local density.

The local density of sample points based on DBSCAN is defined as follows:

**Definition 9** [Local density based on DBSCAN] Given a neighborhood decision system *NS* = (*U, A*∪*d, V, f*), for ∀*x*_*i*_∈*U*, its local density *D*(*x*_*i*_) is defined as follows:


$$\begin{array}{l} D\left(x_{i}\right)=\rho_{x_{i}}\frac{\left|\left\{x_{j\neq i}\in U|L\left(x_{j}\right)=L\left(x_{i}\right)\right\}\right|}{\left|U\right|} \end{array}$$ (12)

Where $\rho_{x_{i}}=\sum_{j\neq i}\exp\left[-{\left(\frac{d_{ij}}{d_{c}}\right)}^{2}\right]$, *d*_*ij*_ is the distance between the *x*_*i*_ and *x*_*j*_, *d*_*c*_ is a constant taken as 1%–2% of the total objects. And *L*(*x*) denotes the label of *x* after processing by the DBSCAN algorithm.

The local density consists of two parts. The first component represents the Gaussian kernel density of sample *x*_*i*_, while the second component denotes the proportion of samples with the same label as *x*_*i*_ relative to the total samples after DBSCAN processing.

After obtaining the local density of samples, we further determine the granularity centers of decision classes:

**Definition 10** [The granularity center] Given a neighborhood decision system *NS* = (*U, A*∪*d, V, f*), *R*_*d*_ induces a partition of *U* as $ℱ=\left\{X_{1},X_{2},...,X_{z}\right\}$. For ∀*X*_*j*_(*j* = 1, 2, ..., *z*), the granularity center *C*_ρ_(*X*_*j*_) of *X*_*j*_ is defined as follows:


$$\begin{array}{l} C_{\rho }\left(X_{j}\right)=\underset{x_{i}\in X_{j}}{\arg\max}\left(D\left(x_{i}\right)\right) \end{array}$$ (13)

Where *D*(*x*_*i*_) is given as in Equation 12. Note that ρ(*X*_*j*_) is the densest sample in *X*_*j*_, not a virtual point.

**Figure 3:**
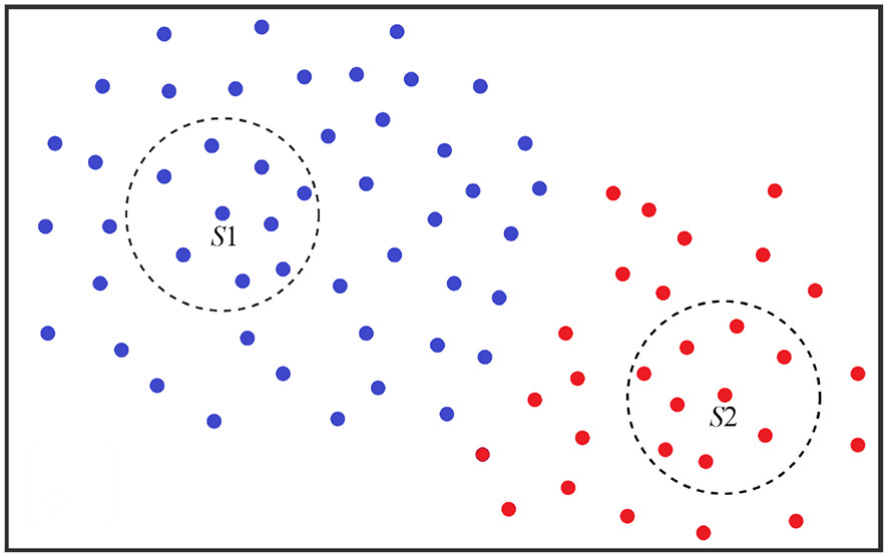
The granulation centers are found using DBSCAN.

As shown in Figure 3, the points *S*_1_ and *S*_2_ possess identical Gaussian kernel density distributions. However, after DBSCAN processing, they are divided into distinct clusters. It is clearly observable that the total number of samples sharing the same label as *S*_1_ (blue samples) exceeds the total number of samples sharing the same label as *S*_2_ (red samples). Considering the overall sample distribution, we select the denser cluster *S*_1_ as the granularity center. This choice is more reasonable and conducive to generating information granularity.

Therefore, we incorporate DBSCAN into interval granulation generation to propose a DBSCAN-based local density interval generation method, obtaining a more reasonable interval granulation center, namely DBIG. This algorithm not only ensures that the output interval particles effectively partition the universe but also overcomes the limitations of GBC. In more detail, firstly, the highest-density cluster centers obtained by the DBSCAN algorithm in the decision class are used as the initial interval granularity centers. Subsequently, expanding outward from granularity centers, finding sample points with identical labels in their surroundings, and determining the upper and lower bounds of intervals based on the values of conditional attributes for these sample points, thereby effectively dividing many real numbers or infinite continuous values into multiple intervals. Finally, interval equivalence relations induce interval granularity, and the process iterates repeatedly until the entire universe is partitioned. In short, the core steps of the DBIG algorithm include: calculating the initial interval granularity centers and generating interval granularity with purity equal to 1 based on granularity centers.

### 4.2 Complexity analysis of DBIG

The computational complexity of the proposed DBIG algorithm is primarily determined by the iterative interval generation process. Since samples that have completed granulation are excluded from subsequent iterations, the computational cost progressively decreases. Consequently, the complexity is dominated by the first iteration, which processes all *n* samples. This iteration consists of two main components:

- **Clustering process:** The standard DBSCAN algorithm has a time complexity of *O*(*n*^2^). However, by employing spatial indexing structures (such as KD-trees) for nearest neighbor search, this can be optimized to approximately *O*(*n*log*n*) in lower-dimensional spaces.
- **Density estimation:** Identifying granularity centers requires computing pairwise Euclidean distances (as defined in Equation 12). This step constitutes the main computational bottleneck with a worst-case complexity of *O*(*n*^2^).

In summary, the overall time complexity of the DBIG algorithm is *O*(*n*^2^). While this is higher than simple linear methods, it is acceptable for offline training. To mitigate computational costs on large-scale datasets, potential strategies include utilizing KD-tree-based accelerated neighbor search or adopting sampling-based approximate density estimation to approach *O*(*n*log*n*) efficiency.

### 4.3 Interval granulation neighborhood rough sets model

Furthermore, to better measure the quality of interval granules generated by DBIG, we propose the quality indicator as follows:

**Definition 11** [Quality indicator] Let *IGs* = {*IG*_1_, *IG*_2_, ⋯ , *IG*_*n*_} be an interval granule space. For any *IG*_*j*_∈*IGs*, its quality indicator is expressed as follows:


$$\begin{array}{l} m\left(IG_{j}\right)=\frac{p\left(IG_{j}\right)\times \left(\rho \left(IG_{j}\right)+1\right)}{2} \end{array}$$ (14)

where *p*(*IG*_*j*_) denotes the purity of *IG*_*j*_ calculated by Equation 5, and ρ(*IG*_*j*_) represents the normalized local density, defined as $\rho \left(IG_{j}\right)=\frac{1}{\left|IG_{j}\right|}\underset{x_{i}\in IG_{j}}{\sum }\exp\left[-{\left(\frac{d_{ij}}{d_{c}}\right)}^{2}\right]$. Here, *d*_*c*_ is a constant taken as 1%-2% of the total samples in *IG*_*j*_, and *d*_*ij*_ represents the distance between the center of *IG*_*j*_ and sample *x*_*i*_ within *IG*_*j*_.

**Rationale:** The design motivation for Equation 14 is to comprehensively evaluate the validity of a granule by balancing **correctness** and **representativeness**.

- **Correctness:** The purity term *p*(*IG*_*j*_) ensures label consistency. A higher purity implies that the interval granule is less likely to contain noise or conflicting labels.
- **Representativeness:** The density term ρ(*IG*_*j*_) reflects the topological importance. Granules located in high-density regions are considered more representative of the core data structure compared to outliers.
- **Smoothing:** The term (ρ(*IG*_*j*_)+1)/2 acts as a smoothing and normalization factor. It ensures that sparse but pure granules are not penalized too heavily (avoiding zero multiplication), while still favoring dense granules. The resulting *m*(*IG*_*j*_) effectively maps the quality to the interval [0, 1], serving as a fuzzy membership degree.

Then, the average fuzzy set of *X* based on interval granules is represented as follows:


$$\begin{array}{l} X_{\mathbb{R}}^{J}=\frac{m\left(IG_{1}\right)}{IG_{1}}+\frac{m\left(IG_{2}\right)}{IG_{2}}+\cdots +\frac{m\left(IG_{n}\right)}{IG_{n}} \end{array}$$ (15)

From Definition 7, we have the definition of the average fuzziness of *IGs* based on Equation 11 as follows:

**Definition 12** [Average fuzziness of *IGs*] Given a neighborhood decision system *NS* = (*U, A*∪*d, V, f*), *IGs* = {*IG*_1_, *IG*_2_, …, *IG*_*n*_} is an interval granule space generated on *NS*. *R*⊆*A* and *X*⊆*U*. The average fuzziness of $X_{\mathbb{R}}^{J}$ is represented by:


$$\begin{array}{l} H_{X_{\mathbb{R}}^{J}}=\frac{1}{\left|U\right|}\sum_{IG\in IGs}\xi (IG) \end{array}$$ (16)

where ξ(*IG*) = 4*m*(*IG*)(1−*m*(*IG*)).

According to Definition 12, the average fuzziness of $X_{\mathbb{R}}^{J}$ essentially reflects the uncertainty of an interval granule space. Therefore, we further define an improved interval granulation neighborhood rough sets (IGNRS) as follows:

**Definition 13** [IGNRS] Given a neighborhood decision system *NS* = (*U, A*∪*d, V, f*), *IGs* = {*IG*_1_, *IG*_2_, …, *IG*_*n*_} is an interval granule space generated on *NS* by DBIG. (β, α) (0 ≤ β < α ≤ 1) denotes a threshold pair. *R*⊆*A* and *X*⊆*U*, ∀*IG*_*j*_∈*IGs*, the lower and upper approximation of *X* with respect to *R* are defined as follows:


$$\begin{array}{l} \underline{G}(X)=\left\{x\in U|x\in IG_{j},m\left(IG_{j}\right)\geq \alpha \right\} \end{array}$$ (17)

$$\begin{array}{ll} \bar{G}\left(X\right) & =\left\{x\in U|x\in IG_{j},m\left(IG_{j}\right)>\beta \right\} \end{array}$$ (18)

Accordingly, the rules for dividing the three decision regions are defined as:


$$\begin{array}{l} \mathbb{P}OS_{R}^{\left(\beta ,\alpha \right)}(X)=\underline{G}(X) \end{array}$$ (19)

$$\begin{array}{l} \mathbb{N}EG_{R}^{\left(\beta ,\alpha \right)}(X)=U-\underline{G}(X) \end{array}$$ (20)

$$\begin{array}{l} B\mathbb{N}D_{R}^{\left(\beta ,\alpha \right)}(X)=\bar{G}(X)-\underline{G}(X) \end{array}$$ (21)

Compared to GBNRS, IGNRS reduces boundary information loss and achieves better universe coverage. It can adaptively adjust the size of interval granules based on data distribution across different attribute dimensions. Furthermore, in combination with DBIG, IGNRS resolves the issue of the inaccuracy of granulation center positioning. By processing and representing samples with higher quality, the robustness of the model is further enhanced.

## 5 Three-way decision with interval granulation neighborhood rough sets (3WD-IGNRS) based on fuzziness

Traditional three-way decision (3WD) partitions a three-way decision neighborhood rough set (3WDNRS) into three regions according to predefined risk parameters, aiming to achieve decision-making with minimum expected cost. The reasonable determination of risk parameters has therefore become a critical research issue in three-way decision theory. Within the general 3WD framework proposed by Yang and Yao (2021), this issue has been investigated from three main perspectives, namely minimum distance, minimum cost, and uncertainty invariance.

Existing interval granulation neighborhood rough set (IGNRS) models primarily adopt a two-way decision strategy, which may lead to increased decision errors and information loss when handling uncertain objects. In particular, compared with corresponding three-way decision classifiers, these two-way schemes are less effective in preserving decision certainty. To address this limitation, this study incorporates fuzzy measures into IGNRS and proposes a three-way classifier based on interval granular neighborhood rough sets, termed 3WD-IGNRS, with adaptively determined threshold pairs. By introducing fuzzy measures, the proposed model effectively reduces decision errors and uncertainty loss induced by two-way decision-making, thereby enhancing the robustness of IGNRS-based classification.

By employing fuzzy–rough transformation, shadowed sets (Pedrycz, 1998; Deng and Yao, 2014) naturally emerge as a three-way approximation of fuzzy sets. Unlike approaches that rely on subjective experience, the lower and upper threshold pairs used to construct shadowed sets are automatically derived from the data, thus avoiding potential bias caused by arbitrarily specified thresholds. Specifically, objects whose membership degrees lie between the lower and upper thresholds are assigned to the shadowed region. Under this mechanism, the value domain is extended to an uncertainty interval [β^*^, α^*^], enabling the construction of three-way approximations with shadowed sets (3WA-SS) based on interval granules. Following the formulation in Yang et al. (2024), the principle of 3WA-SS is formally defined as follows.

**Definition 14** [Three-way approximations with shadowed sets (3WA-SS) (Yang et al., 2024)] Given a neighborhood decision system *NS* = (*U, A*∪*d, V, f*), let *IGs* = {*IG*_1_, *IG*_2_, …, *IG*_*n*_} denote an interval granule space generated on *NS*. For *R*⊆*A* and *X*⊆*U*, a mapping $\psi :X_{R}^{J}\to \left\{0,m,1\right\}$ is defined to represent the three-way approximation of $X_{R}^{J}$ using a shadowed set. Specifically, the mapping ψ is defined as


$$\begin{array}{l} \psi \left(X_{R}^{J}\right)=\{\begin{array}{ll} 0, & m\left(IG_{j}\right)\leq \beta^{*},\\ m, & \beta^{*}<m\left(IG_{j}\right)<\alpha^{*},\\ 1, & m\left(IG_{j}\right)\geq \alpha^{*}, \end{array} \end{array}$$ (22)

Where *j* = 1, 2, …, *n*, and *m*(*IG*_*j*_) denotes the membership degree of interval granule *IG*_*j*_.

According to Definition 14, the boundary region of an interval shadowed set is illustrated as an uncertainty interval rather than a crisp boundary. The corresponding threshold pair is determined in a data-driven manner, thereby enhancing both rationality and interpretability.

First, the average membership degree of each interval granule *IG*_*j*_ (*IG*_*j*_∈*IGs*) is calculated to construct an average fuzzy set over the interval granule space $X_{R}^{J}$. Subsequently, a three-way approximation $\psi \left(X_{R}^{J}\right)$ is applied to $X_{R}^{J}$ according to the following rules.

When $m\left(IG_{j}\right)\leq \beta^{*}$, the membership degree *m*(*IG*_*j*_) is reduced to 0, indicating that *IG*_*j*_ is assigned to the negative region, where fuzziness is completely eliminated. When $m\left(IG_{j}\right)\geq \alpha^{*}$, the membership degree *m*(*IG*_*j*_) is elevated to 1, indicating that *IG*_*j*_ is assigned to the positive region, which is also free of fuzziness. When $\beta^{*}<m\left(IG_{j}\right)<\alpha^{*}$, the membership degree is transformed into an intermediate value *m*, and *IG*_*j*_ is assigned to the boundary region, where uncertainty is preserved.

**Figure 4:**
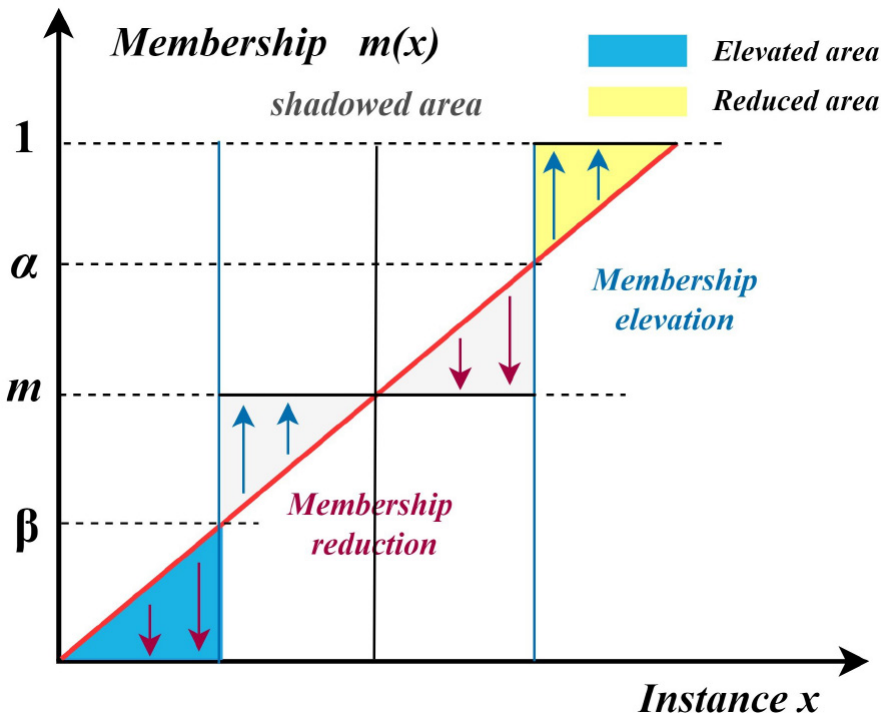
Illustration of three-way approximations with shadowed sets. The horizontal axis represents the instances x, and the vertical axis represents the membership degree m(x).

In the case $\beta^{*}<m\left(IG_{j}\right)<\alpha^{*}$, the interval [β^*^, α^*^] corresponds to the boundary region characterized by significant uncertainty. As illustrated in Figure 4, the upward arrow denotes the elevation operation, whereas the downward arrow represents the reduction operation. Since the positive and negative regions are crisp, their fuzziness values are zero, i.e.,


$$H_{\psi \left(X_{R}^{J}\right)}\left(\text{POS}\right)=H_{\psi \left(X_{R}^{J}\right)}\left(\text{NEG}\right)=0.$$

Consequently, the total fuzziness of the shadowed set $\psi \left(X_{R}^{J}\right)$ is solely determined by the boundary region and is computed as


$$\begin{array}{l} H_{\psi \left(X_{R}^{J}\right)}=H_{\psi \left(X_{R}^{J}\right)}(\text{BND})=T\int_{\beta^{*}}^{\alpha^{*}}\bar{\mu }(x)(1-\bar{\mu }(x))dx, \end{array}$$ (23)

where $T=\frac{4|\left\{x\in U|\beta^{*}<\bar{\mu }\left(x\right)<\alpha^{*}\right\}|}{\left|U\right|}$ and $\bar{\mu }\left(x\right)$ denotes the average membership degree of object *x*.

As reported in Zhang et al. (2013) and Wu and Mendel (2009), uncertainty measurement plays a crucial role in decision systems. Objectively evaluating fuzziness without relying on subjective expert knowledge significantly improves the credibility of the decision process. Moreover, preserving fuzziness invariance is essential for minimizing fuzziness loss. Therefore, by integrating Equations 16, 23, an objective function is constructed to determine optimal thresholds based on the principle of fuzziness invariance.

According to the derivation in Yang et al. (2024), the threshold pair (α^*^, β^*^) can be obtained from the parameter *m* as


$$\alpha^{*}=\frac{1+\sqrt{1-2m\left(1-m\right)}}{2},\;\beta^{*}=\frac{1-\sqrt{1-2m\left(1-m\right)}}{2}.$$

Let *m*_1_ and *m*_2_ be two constants satisfying 0 < *m*_1_ ≤ 0.5, 0.5 ≤ *m*_2_ < 1, and *m*_1_+*m*_2_ = 1. The above expressions can then be rewritten as


$$\alpha^{*}=\frac{1+\sqrt{1-2\left(m_{1}m_{2}\right)}}{2},\;\beta^{*}=\frac{1-\sqrt{1-2\left(m_{1}m_{2}\right)}}{2}.$$

The optimal threshold pair is obtained by solving the following optimization problem:


$$\begin{array}{l} \underset{0\leq m_{1}\leq m_{2}\leq 1}{\mathrm{argmin}}|{\bar{H}}_{\psi \left(X_{R}^{J}\right)}-{\bar{H}}_{X_{R}^{J}}| \end{array}$$ (24)

$$\begin{array}{l} \text{s.t.}\;0\leq m_{1}\leq 0.5,\;0.5\leq m_{2}\leq 1, \end{array}$$ (25)

where


$$\begin{array}{l} {\bar{H}}_{\psi \left(X_{R}^{J}\right)}-{\bar{H}}_{X_{R}^{J}}=H_{\psi \left(X_{R}^{J}\right)}\left(\text{BND}\right)-\frac{1}{\left|U\right|}\underset{IG\in IGs}{\sum }\xi \left(IG\right). \end{array}$$ (26)

The detailed threshold acquisition procedure is summarized in Algorithm 1. By minimizing the above objective function, the optimal threshold pair and the corresponding three regions can be obtained, thereby minimizing fuzziness loss. In contrast, employing arbitrary thresholds would produce different region partitions and inevitably lead to increased uncertainty loss. Based on the principle of 3WA-SS, we further develop a three-way classifier using interval granular neighborhood rough sets, termed 3WD-IGNRS, which is introduced in the following subsection.

Algorithm 1. **Threshold acquisition process.**

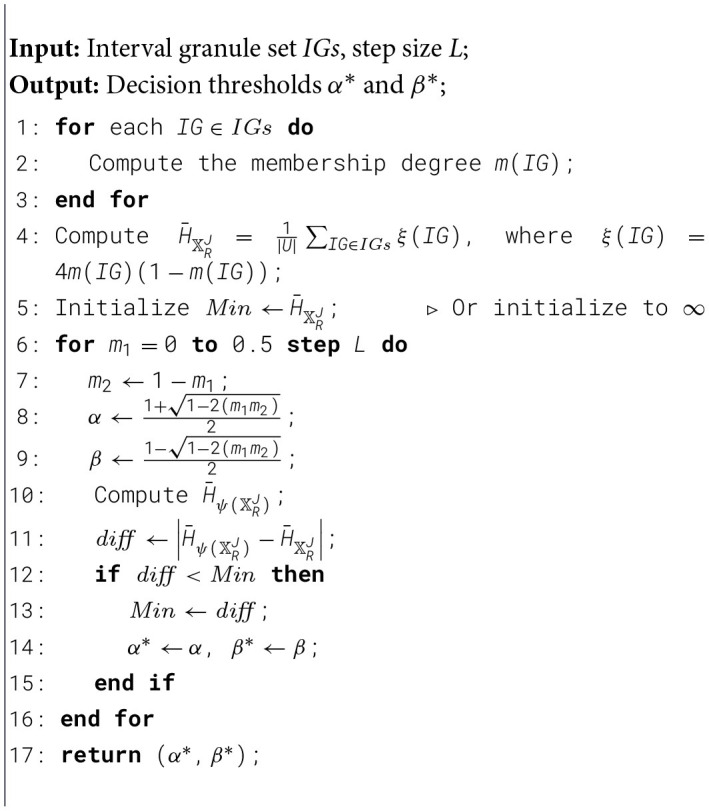

## 6 Experimental studies

In this section, we conduct a comprehensive experimental evaluation of the proposed 3WD-IGNRS by comparing it with three state-of-the-art granular-ball (GB)-based classifiers: GBKNN (Xia et al., 2019), ACC-GBKNN (Xia et al., 2022), and GBKNN++ (Xie et al., 2024). Additionally, four widely used traditional machine learning classifiers are included as baselines: *k*-Nearest Neighbors (KNN), Support Vector Machines (SVM), Normalized Cross-Correlation (NC)-based classification, and Classification and Regression Trees (CART).

The experimental evaluation is designed to assess the performance from four key perspectives: (1) **Fuzziness comparison**, to analyze the uncertainty loss during granulation; (2) **Effectiveness analysis**, validating classification metrics against baselines; (3) **Robustness analysis**, testing performance under varying noise ratios (10%–40%); and (4) **Stability analysis**, evaluating performance fluctuations.

### 6.1 Datasets and preprocessing

All experiments are conducted on 14 benchmark datasets, including 11 from the UCI repository (UCI Machine Learning Repository, 2024) and 3 from the KEEL repository (Alcalá-Fdez et al., 2011). These datasets cover a wide range of dimensions and sample sizes to fully test the model's generalization ability. The detailed characteristics are summarized in Table 2.

**Table 2:** The details of experimental datasets.

ID	Dataset	Attributes	Instances	Type	Source
*D*_1_	Breast-cancer	9	699	Integer	UCI
*D*_2_	Cloud	10	1,024	Real	UCI
*D*_3_	Airfoil self-noise	5	1,503	Real	UCI
*D*_4_	Car	6	1,728	Categorical	KEEL
*D*_5_	Wifi_localization	7	2,000	Real	UCI
*D*_6_	Segment	19	2,310	Real	UCI
*D*_7_	Phoneme	5	5,404	Real, Integer	KEEL
*D*_8_	Shill bidding	9	6,321	Real	UCI
*D*_9_	Satimage	36	6,435	Integer	UCI
*D*_10_	Twonorm	20	7,400	Integer, Real	KEEL
*D*_11_	Electrical	13	10,000	Real	UCI
*D*_12_	Dry bean	16	13,611	Integer, Real	UCI

All UCI datasets are obtained from the UCI machine learning repository, while KEEL datasets are collected from the KEEL repository (UCI Machine Learning Repository, 2024; Alcalá-Fdez et al., 2011).

**Data preprocessing:** since the proposed DBIG algorithm relies on a distance metric for density estimation, appropriate data preprocessing is essential.

- **Continuous attributes:** We applied Min–Max normalization to scale all continuous feature values into the range [0, 1], eliminating the influence of different attribute scales.
- **Categorical attributes:** For datasets containing categorical variables (e.g., *Breast-cancer, Car*, and *Mushroom*), we employed **Label encoding** to transform non-numerical labels into numerical values. This transformation enables the valid computation of Euclidean distances required by the DBSCAN clustering process within our framework.

### 6.2 Experimental setup and metrics

A 10-fold cross-validation strategy is adopted for each dataset to ensure the reliability of the results. All experiments are implemented on a platform equipped with an Intel Core i5-4210M processor (2.60 GHz), 8.0 GB DDR3 memory, running a 64-bit Windows 10 operating system with Python 3.10.

To quantitatively evaluate the classification performance, four standard metrics are employed: *Accuracy*, *Precision*, *Recall*, and *F*_1_-score. Furthermore, to verify the statistical significance of the results, we report:

- ***Win/loss*:** the count of datasets where 3WD-IGNRS outperforms vs. underperforms the competing methods.
- ***Rank*:** the average ranking of each algorithm across all datasets (lower is better).
- **p*-values*:** calculated using the Wilcoxon signed-rank test. A *p*-value less than 0.05 indicates a statistically significant difference between 3WD-IGNRS and the comparison method.

The detailed statistical results are presented in the following subsections.

In this section, we conduct a comprehensive evaluation of the proposed 3WD-IGNRS by comparing it with three state-of-the-art GB-based classifiers, namely GBKNN (Xia et al., 2019), ACC-GBKNN (Xia et al., 2022), and GBKNN++ (Xie et al., 2024), as well as four traditional machine learning classifiers: *k*-Nearest Neighbors (KNN), Support Vector Machines (SVM), Normalized Cross-Correlation (NC), and Classification and Regression Trees (CART). The experimental evaluation is carried out from the following four perspectives:

To facilitate a clearer understanding of the experimental setup, the hyperparameter configurations of the comparative methods are briefly summarized as follows:

- **GBKNN, ACC-GBKNN, and GBKNN++:** As higher purity levels allow GBs to represent the original dataset more accurately, the purity threshold for all GB-based classifiers is set to 1.0 to ensure high descriptive precision without sacrificing generalization ability. In addition, the neighborhood size *k* in the nearest-neighbor classification stage is set to 1 for all GB-based methods.
- **KNN:** The neighborhood size *k* is selected from the set {1, 3, 5, 7, 9, 11, 13, 15}, and the optimal value is determined based on classification performance.
- **SVM:** The radial basis function (RBF) kernel is adopted, and the kernel parameter is set as the reciprocal of the total number of features in each dataset.
- **NC and CART:** All hyperparameters are kept consistent with the default settings provided by the *scikit-learn* library.

### 6.3 Parameter settings

In our experiments, the hyperparameters for DBSCAN (Epsilon ϵ and MinPts) were determined based on the distribution of each dataset. Typically, MinPts was set to 4. For Algorithm 1, the step size *L* was set to 0.01 to ensure a balance between precision and computational efficiency.

### 6.4 Fuzziness comparison: IGNRS vs. 3WD-IGNRS

**Figure 5:**
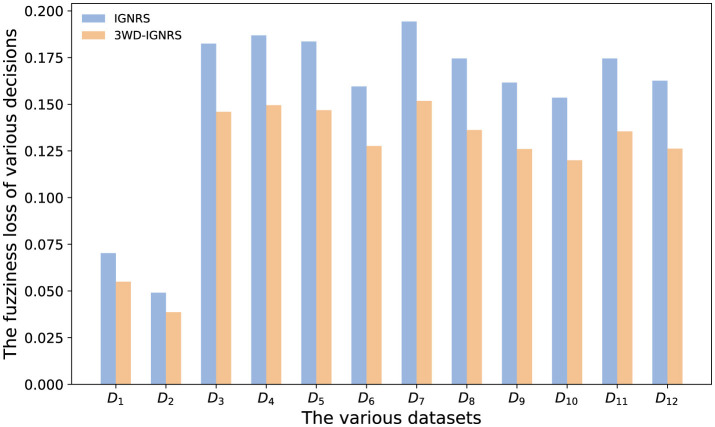
Comparison of quantitative fuzziness loss between IGNRS and 3WD-IGNRS across 12 benchmark datasets (*D*_1_–*D*_12_). The *x*-axis represents the dataset indices, and the *y*-axis represents the calculated fuzziness loss value. The blue bars denote the baseline IGNRS, while the orange bars denote the proposed 3WD-IGNRS. A lower value indicates reduced decision uncertainty.

To empirically validate the effectiveness of the proposed uncertainty invariance principle, we quantitatively evaluated the fuzziness loss strictly following the objective function defined in Equation 26. The comparison results between the original IGNRS model (based on two-way decisions) and the proposed 3WD-IGNRS model are visualized in Figure 5.

As shown in Figure 5, 3WD-IGNRS consistently yields lower fuzziness loss values compared to IGNRS across all 12 datasets. For instance, significant reductions are observed in datasets *D*_1_ and *D*_7_. This improvement can be attributed to the structural advantage of the three-way decision mechanism. Unlike IGNRS, which forces uncertain interval granules into positive or negative regions (thereby increasing information entropy and decision risk), 3WD-IGNRS utilizes a **boundary region** to buffer these granules. By deferring decisions for high-uncertainty samples, the proposed model minimizes the global fuzziness loss, effectively preserving the uncertainty invariance of the granular structure throughout the classification process.

### 6.5 Efficiency analysis

**Figure 6:**
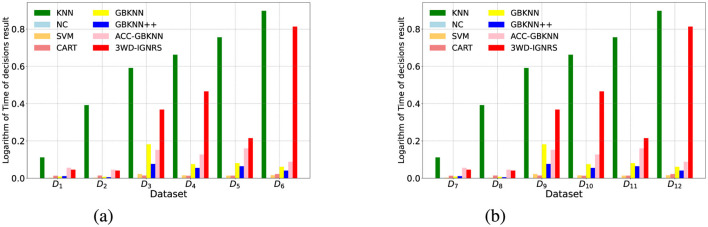
Comparison of the logarithm of running times across 12 datasets. The *y*-axis represents the base-10 logarithm of the total execution time (seconds). Standard KNN exhibits exponential time growth on larger datasets, whereas GB-based methods, including the proposed 3WD-IGNRS, maintain high scalability. **(a)** Datasets *D*_1_–*D*_6_. **(b)** Datasets *D*_7_–*D*_12_.

To empirically evaluate the computational efficiency of the proposed framework, we recorded the total running time (in seconds) of all eight algorithms across the 12 benchmark datasets. Because the running times span multiple orders of magnitude (e.g., standard KNN suffers from severe scalability issues on large datasets), we visualized the base-10 logarithm of the running times to facilitate a clearer comparison. The results are presented in Figure 6.

As clearly observed in Figure 6, the standard KNN algorithm exhibits prohibitive time complexity on larger datasets (e.g., *D*_11_ and *D*_12_), making it unsuitable for large-scale applications. In contrast, the granular-ball-based methods significantly compress the sample space, resulting in much faster execution.

It is worth noting that the running time of the proposed 3WD-IGNRS is slightly higher than that of extreme-fast baseline methods (such as NC or CART) and some simplified GB variants (like GBKNN++). This is well within expectations. The additional time overhead in 3WD-IGNRS is primarily consumed during the offline training phase by two crucial steps: (1) the DBIG interval granulation process designed to capture accurate topological boundaries, and (2) the objective optimization process (Algorithm 1) for finding the optimal three-way decision thresholds based on uncertainty invariance. Considering the substantial improvements in classification accuracy, fuzziness reduction, and noise robustness demonstrated in the preceding sections, this slight increase in computational time is a highly worthwhile and acceptable trade-off for complex decision-making scenarios.

### 6.6 Effectiveness of 3WD-IGNRS

This experiment compares seven classification algorithms, including KNN, NC, SVM, CART, GBKNN, ACC-GBKNN, and GBKNN++. As reported in Table 3, the performance of all methods is evaluated using four metrics: *Accuracy*, *Precision*, *Recall*, and *F*1-*score*. The average values of these metrics over the 12 datasets are presented in the penultimate row of Table 3. The results show that 3WD-IGNRS achieves the highest average performance across all four metrics, indicating its superior generalization ability.

**Table 3:** The statistical analysis of various algorithms.

No.	Metrics	KNN	NC	SVM	CART	GBKNN	GBKNN++	ACC-GBKNN	3WD-IGNRS
*D*_1_	*Accuracy*	0.9386	0.9277	0.9407	0.7810	0.9471	0.9346	0.9325	0.9777
	*F*1 *score*	0.9328	0.9245	0.9294	0.7497	0.9321	0.9340	0.9319	0.9695
	*Recall*	0.9397	0.9089	0.9600	0.7166	0.9871	0.9288	0.9417	0.9911
	*Precision*	0.9392	0.9530	0.9203	0.8273	0.9019	0.9492	0.9344	0.9588
*D*_2_	*Accuracy*	0.9883	0.8941	0.9854	0.9197	0.9647	0.9863	0.9912	0.9980
	*F*1 *score*	0.9941	0.9275	0.9925	0.9381	0.9798	0.6132	0.7228	0.9990
	*Recall*	0.9893	0.9999	0.9912	0.9932	0.9961	0.9980	0.9990	0.9980
	*Precision*	0.9990	0.8924	0.9940	0.9250	0.9680	0.9882	0.9922	0.9999
*D*_3_	*Accuracy*	0.8499	0.6982	0.8352	0.7480	0.8319	0.5600	0.5310	0.9209
	*F*1 *score*	0.8943	0.7518	0.8795	0.7862	0.8763	0.5045	0.4810	0.9512
	*Recall*	0.8605	0.8128	0.8605	0.8549	0.8687	0.6142	0.5617	0.9216
	*Precision*	0.9425	0.7116	0.9327	0.7786	0.9018	0.7146	0.7040	0.9888
*D*_4_	*Accuracy*	0.9554	0.8512	0.9635	0.8917	0.9305	0.9138	0.9144	0.9999
	*F*1 *score*	0.9677	0.8831	0.9739	0.9298	0.9521	0.8974	0.8909	0.9999
	*Recall*	0.9807	0.9611	0.9793	0.9036	0.9305	0.9338	0.9595	0.9999
	*Precision*	0.9554	0.8215	0.9694	0.9661	0.9760	0.9442	0.9257	0.9999
*D*_5_	*Accuracy*	0.9730	0.7765	0.9715	0.876	0.9765	0.9635	0.9755	0.9914
	*F*1 *score*	0.9756	0.7629	0.9739	0.8544	0.9776	0.9634	0.9755	0.9934
	*Recall*	0.9643	0.8470	0.9638	0.9262	0.9713	0.9700	0.9880	0.9888
	*Precision*	0.9910	0.7760	0.9880	0.8740	0.9860	0.9604	0.9660	0.9985
*D*_6_	*Accuracy*	0.9928	0.6569	0.9966	0.9756	0.9731	0.9899	0.9866	0.9985
	*F*1 *score*	0.9745	0.4604	0.9881	0.9363	0.8801	0.9798	0.9730	0.9949
	*Recall*	0.9805	0.3045	0.9966	0.9257	1.000	0.9798	0.9666	0.9903
	*Precision*	0.9697	0.9794	0.9799	0.9663	0.8124	0.9652	0.9447	0.9999
*D*_7_	*Accuracy*	0.7936	0.7089	0.7906	0.5766	0.7951	0.8598	0.8734	0.9207
	*F*1 *score*	0.6495	0.6332	0.6650	0.3426	0.6524	0.8274	0.8472	0.6680
	*Recall*	0.7253	0.6444	0.7146	0.4205	0.7084	0.7321	0.7872	0.8309
	*Precision*	0.6359	0.7590	0.6814	0.3859	0.6615	0.7755	0.7810	0.6111
*D*_8_	*Accuracy*	0.9862	0.9713	0.9794	0.9943	0.9818	0.9840	0.9843	0.9964
	*F*1 *score*	0.9438	0.8968	0.9134	0.9750	0.9248	0.9591	0.9596	0.9810
	*Recall*	0.9367	0.8388	0.8818	0.9709	0.9016	0.9526	0.9452	0.9774
	*Precision*	0.9614	0.9851	0.9570	0.9807	0.9599	0.9040	0.9117	0.9856
*D*_9_	*Accuracy*	0.9832	0.7125	0.9800	0.9484	0.9737	0.9863	0.9809	0.9955
	*F*1 *score*	0.9634	0.6509	0.9552	0.8478	0.9424	0.9815	0.9744	0.9884
	*Recall*	0.9673	0.5991	0.9627	0.9326	0.9628	0.9804	0.9706	0.9945
	*Precision*	0.9641	0.8115	0.9549	0.8557	0.9270	0.9652	0.9545	0.9836
*D*_10_	*Accuracy*	0.9718	0.9753	0.9731	0.7036	0.9680	0.9469	0.9561	0.9926
	*F*1 *score*	0.9716	0.9751	0.9729	0.6549	0.9679	0.9469	0.9561	0.9928
	*Recall*	0.9738	0.9772	0.9757	0.6787	0.9688	0.9516	0.9613	0.9934
	*Precision*	0.9702	0.9737	0.9710	0.6822	0.9675	0.9428	0.9513	0.9922
*D*_11_	*Accuracy*	0.9127	0.8686	0.9747	0.9998	0.8861	0.8619	0.8450	0.9904
	*F*1 *score*	0.8686	0.8339	0.9657	0.9997	0.8355	0.8513	0.8355	0.9799
	*Recall*	0.9446	0.7886	0.9672	0.9997	0.8774	0.8227	0.8351	0.9903
	*Precision*	0.8094	0.9006	0.9663	0.9997	0.8050	0.8017	0.7605	0.9700
*D*_12_	*Accuracy*	0.9759	0.8548	0.9804	0.9442	0.9677	0.9440	0.9505	0.9901
	*F*1 *score*	0.9205	0.7653	0.9326	0.7977	0.8943	0.9058	0.9117	0.9595
	*Recall*	0.9248	0.7072	0.9494	0.8427	0.9013	0.9007	0.9007	0.9774
	*Precision*	0.9270	0.9581	0.9221	0.8255	0.9039	0.8163	0.8226	0.9477
*Average*	*Accuracy*	0.9435	0.8247	0.9476	0.8632	0.9300	0.9101	0.9109	**0.9794**
	*F*1 *score*	0.9214	0.7888	0.9285	0.8177	0.9013	0.8716	0.8637	**0.9536**
	*Recall*	0.9323	0.7825	0.9336	0.8471	0.9228	0.9014	0.8971	**0.9698**
	*Precision*	0.9221	0.8768	0.9364	0.8389	0.8976	0.8874	0.8939	**0.9507**
Statistics	*Win*/*Loss*	47/1	45/3	46/2	44/4	46/2	45/3	45/3	318/18
	*P*−*vlaue*	2.4570E-06	4.2564E-11	1.3679E-05	6.3807E-08	6.3807E-08	3.8112E-08	1.9472E-08	
	*Rank*	**5.2604 (2)**	2.7708	**5.1354 (3)**	3.0833	4.2396	3.9792	3.8958	**7.6354 (1)**

Bold values indicate the best performance.

Furthermore, statistical analyses are conducted using the Friedman test, the Wilcoxon signed-rank test, and average ranking analysis. The corresponding statistical results, including *Win*/*Loss*, *P*-values, and *Rank*, are summarized in the last row of Table 3. Out of 336 *Win*/*Loss* comparisons with competing algorithms, 3WD-IGNRS outperforms its counterparts in 318 cases. The obtained *P*-values are all below 0.05, indicating statistically significant performance differences between 3WD-IGNRS and the comparison methods. In terms of average ranking, 3WD-IGNRS consistently ranks first, followed by KNN and the traditional SVM classifier.

The superior effectiveness of 3WD-IGNRS can be primarily attributed to the following key factors:

1. Compared with four traditional machine learning classifiers, 3WD-IGNRS inherits the intrinsic advantages of information granularity-based classification. By using information granules instead of individual samples as model inputs, the complexity of the learning process is substantially reduced, while robustness against noisy data is significantly enhanced.
2. Compared with existing GB-based classifiers, 3WD-IGNRS adopts the proposed DBIG strategy to construct interval granules. This interval-based representation enables a more expressive and flexible description of data uncertainty, thereby improving the quality of the generated granules.
3. 3WD-IGNRS employs more rational decision rules for selecting interval granular neighborhoods during test sample classification. By integrating three-way decision theory, the proposed method effectively alleviates decision uncertainty and consequently achieves substantial improvements in classification performance.

It is worth noting that while 3WD-IGNRS outperforms comparison methods on most metrics, a specific trade-off was observed on the *Phoneme* dataset (*D*_7_). As shown in Table 3, although the *Precision* of 3WD-IGNRS (0.6111) is lower than that of SVM (0.6814), its *Recall* (0.8309) and overall *Accuracy* (0.9207) are significantly higher (SVM: Recall 0.7146, Accuracy 0.7906).

This phenomenon can be interpreted through the lens of decision strategies. SVM relies on a strict hyperplane to separate classes; on datasets with overlapping boundaries like *Phoneme*, it tends to be conservative, rejecting ambiguous positive samples to maintain high precision, which inevitably sacrifices recall. In contrast, 3WD-IGNRS leverages the **boundary region** to buffer uncertain samples. By utilizing fuzzy membership degrees and deferred decision logic, our model successfully captures a larger number of positive samples that are missed by SVM. Although this strategy introduces a small number of false positives (resulting in slightly lower precision), the substantial gain in recall leads to a marked improvement in overall classification accuracy, demonstrating the effectiveness of 3WD in handling complex, overlapping data distributions.

### 6.7 Robustness and stability of 3WD-IGNRS

To evaluate the robustness of 3WD-IGNRS, artificial label noise was introduced into the datasets with ratios of 10%, 20%, 30%, and 40%, following the procedure outlined in the experimental setup.

**Figure 7:**
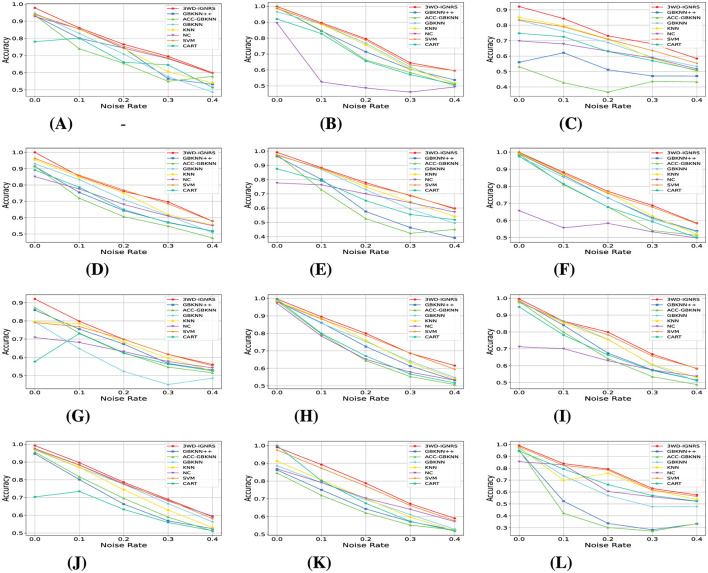
The comparison of accuracy under different noise rates on 12 datasets **(A–L)**. The *x*-axis represents noise rates, and the *y*-axis represents classification accuracy. The red line with circle markers denotes the proposed 3WD-IGNRS, which consistently demonstrates superior robustness compared to other GB-based classifiers across most datasets. **(A)** Breast-cancer. **(B)** Cloud. **(C)** Airfoil. **(D)** Car **(E)** Wifi_Loc. **(F)** Segment. **(G)** Phoneme. **(H)** Shill bidding. **(I)** Satimage. **(J)** Twonorm. **(K)** Electrical. **(L)** Dry Bean.

**Figure 8:**
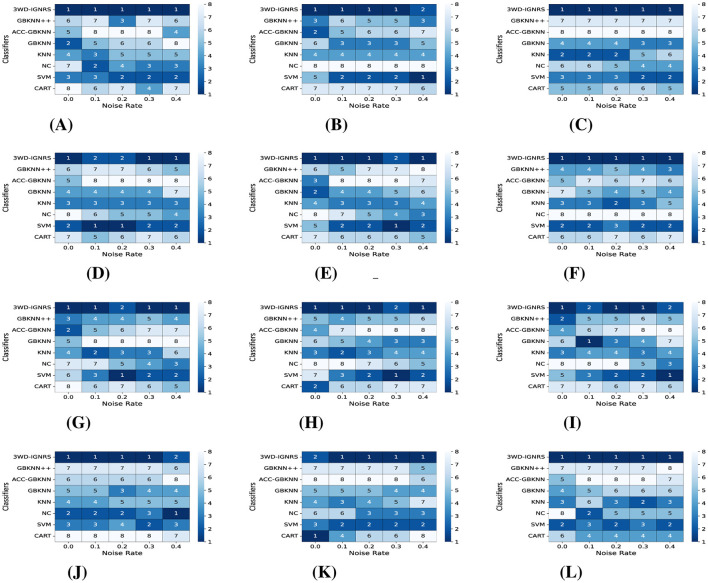
Heatmap visualization of accuracy rankings for different algorithms under varying noise rates across datasets **(A–L)**. Darker blue cells indicate the best performance, while lighter cells indicate lower rankings. 3WD-IGNRS maintains the highest ranking (darkest blue) in the majority of noise scenarios, highlighting its stability. **(A)** Breast-cancer. **(B)** Cloud. **(C)** Airfoil. **(D)** Car **(E)** Wifi_Loc. **(F)** Segment. **(G)** Phoneme. **(H)** Shill bidding. **(I)** Satimage. **(J)** Twonorm. **(K)** Electrical. **(L)** Dry Bean.

For a more intuitive illustration of the experimental results, the classification performance under different noise levels is visualized in Figures 7, 8. Specifically, Figure 7 presents the variation trends of *Accuracy* for GB-based classifiers on each dataset under increasing noise ratios using line plots. As the noise level increases, the *Accuracy* of all methods decreases to varying degrees across different datasets. Nevertheless, in the majority of cases, the performance curve of 3WD-IGNRS consistently remains at the highest position, indicating that it preserves superior classification accuracy even under severe noise interference.

Furthermore, Figure 8 employs heat maps to illustrate the ranking of *Accuracy* for each algorithm on different datasets under various noise ratios. For example, as shown in Figure 7A, on the Breast-Cancer dataset with a noise ratio of 10%, 3WD-IGNRS achieves the highest *Accuracy*, and thus is assigned rank 1 in Figure 8A. Overall, 3WD-IGNRS ranks first on most datasets under noise ratios of 10%, 20%, 30%, and 40%, respectively, demonstrating its strong robustness and stability in noisy environments.

The outstanding noise resistance of 3WD-IGNRS primarily stems from its integration with DBIG and 3WD. First, this combination ensures more precise identification of density centers across all categories. These centers serve as initial points for the interval granularization process, facilitating the generation of larger information granules and significantly enhancing the classifier's robustness against noise. Second, the label of each interval granule is determined by the majority opinion of samples within the interval, effectively reducing the impact of noisy samples and further enhancing robustness. Finally, the delayed decision strategy of 3WD also significantly improves the robustness of 3WD-IGNRS.

### 6.8 The stability of 3WD-IGNRS

We comprehensively evaluated the stability differences among 3WD-IGNRS, GBKNN, ACC-GBKNN, GBKNN++, KNN, NC, SVM, and CART by jointly considering the average, minimum, and maximum classification accuracy. Throughout the entire evaluation process, the data partitioning scheme was kept strictly consistent to ensure fairness and reproducibility.

**Figure 9:**
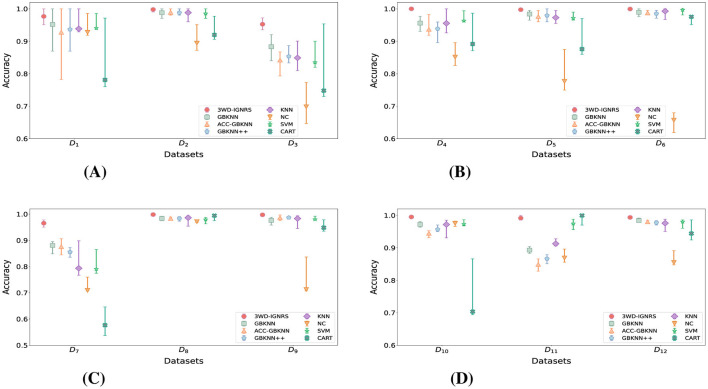
Stability comparison of GB-based classifiers and 3WD-IGNRS. **(A)** Stability on 1st group. **(B)** Stability on 2nd group. **(C)** Stability on 3rd group. **(D)** Stability on 4th group.

The experimental results are illustrated using error bars in Figure 9. In each subfigure, markers with different shapes denote the average test accuracy of each classifier, while the upper and lower horizontal bars indicate the maximum and minimum accuracy values obtained during the 10-fold cross-validation procedure. A narrower error bar implies smaller performance variation and thus higher classification stability. As shown in Figure 9, 3WD-IGNRS consistently exhibits smaller accuracy fluctuations and superior stability compared with the other GB-based classifiers across most datasets.

The superior stability of the proposed 3WD-IGNRS can be primarily attributed to its deterministic interval granulation mechanism and three-way decision framework. Unlike GBKNN and ACC-GBKNN, which rely on random selection of center points during the granular ball splitting process and thus introduce inherent variability, 3WD-IGNRS avoids random parameters entirely. Although GBKNN++ eliminates randomness by fixing the purity threshold, it inevitably discards certain boundary information during granule construction, which may lead to unstable decisions near class boundaries. In contrast, 3WD-IGNRS preserves the complete data space through interval granules and explicitly models boundary uncertainty via three-way decisions, resulting in more consistent and reliable classification outcomes.

**Figure 10:**
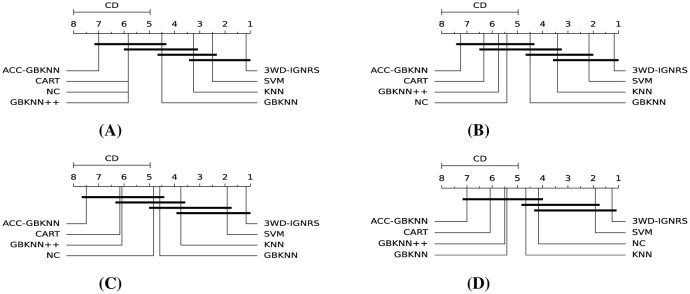
Nemenyi *post-hoc* test under different noise rates. **(A)** Noise rate: 0.1. **(B)** Noise rate: 0.2. **(C)** Noise rate: 0.3. **(D)** Noise rate: 0.4.

Finally, a Nemenyi *post-hoc* test was performed to further investigate the statistical significance of performance differences among the compared classifiers under varying noise conditions. This test determines whether the observed differences in average ranks exceed the critical distance (CD), thereby identifying statistically significant superiority relationships. As illustrated in Figure 10, 3WD-IGNRS consistently outperforms the competing methods with statistically significant margins across different noise levels, further confirming its superior robustness and stability in noisy environments.

## 7 Conclusion

The proposed 3WD-IGNRS is particularly suitable for risk-sensitive applications, such as medical diagnosis and fault detection. In these fields, the delayed decision-making allows the system to request further expert examination for uncertain cases, rather than making a forced, incorrect classification, thereby reducing the cost of misdiagnosis.

To enhance the performance of the three-way decision neighborhood rough set (3WDNRS) framework, this study proposed 3WD-IGNRS by integrating interval granulation neighborhood rough sets with three-way decision theory under the principle of minimizing fuzziness loss. Experimental results demonstrate that 3WD-IGNRS effectively alleviates fuzziness loss during the classification process and consistently outperforms the original IGNRS model. Moreover, extensive comparative experiments conducted on 12 public benchmark datasets show that 3WD-IGNRS achieves superior or highly competitive performance in terms of effectiveness, robustness, and stability when compared with three state-of-the-art GB-based classifiers and four classical machine learning classifiers.

These results indicate that the proposed framework provides a promising and solid foundation for future research on advancing three-way decision theory toward enhanced robustness and generalization capability. Nevertheless, several limitations of the current study should be acknowledged:

- Compared with existing GB-based classifiers, the interval granule generation process in 3WD-IGNRS is relatively computationally demanding and requires further optimization to improve efficiency.
- The current framework lacks a multilevel or hierarchical extension of sequential 3WD-IGNRS, as well as an optimal interval granule space selection strategy for progressive decision-making.

Accordingly, future research will focus on the following directions:

- Developing an incremental learning version of 3WD-IGNRS to support online feature selection, by explicitly modeling the dynamic updating mechanism of interval granules.
- Further improving the efficiency of interval granule generation by incorporating unsupervised GB-based learning strategies. In addition, two key granularity rationality measures–coverage and specificity–will be introduced as optimization criteria to construct an optimal interval granularity space selection mechanism.
- Extending the current interval granulation method to effectively handle ultra-high-dimensional datasets, potentially by integrating subspace clustering or feature selection mechanisms to overcome the curse of dimensionality.

## Data Availability

The original contributions presented in the study are included in the article/supplementary material, further inquiries can be directed to the corresponding author.
